# Effect of Tea Polyphenol Compounds on Anticancer Drugs in Terms of Anti-Tumor Activity, Toxicology, and Pharmacokinetics

**DOI:** 10.3390/nu8120762

**Published:** 2016-12-14

**Authors:** Jianhua Cao, Jie Han, Hao Xiao, Jinping Qiao, Mei Han

**Affiliations:** 1 Key Laboratory of Radiopharmaceuticals, Ministry of Education, College of Chemistry, Beijing Normal University, Beijing 100875, China; caojianhua0303@163.com (J.H.C.); 201521150080@mail.bnu.edu.cn (H.X.); 2 Analytical Center, Beijing Normal University, Beijing 100875, China; 13701290930@139.com

**Keywords:** tea polyphenol, anticancer agent, synergistic anticancer activity, toxicology, pharmacokinetics

## Abstract

Multidrug resistance and various adverse side effects have long been major problems in cancer chemotherapy. Recently, chemotherapy has gradually transitioned from mono-substance therapy to multidrug therapy. As a result, the drug cocktail strategy has gained more recognition and wider use. It is believed that properly-formulated drug combinations have greater therapeutic efficacy than single drugs. Tea is a popular beverage consumed by cancer patients and the general public for its perceived health benefits. The major bioactive molecules in green tea are catechins, a class of flavanols. The combination of green tea extract or green tea catechins and anticancer compounds has been paid more attention in cancer treatment. Previous studies demonstrated that the combination of chemotherapeutic drugs and green tea extract or tea polyphenols could synergistically enhance treatment efficacy and reduce the adverse side effects of anticancer drugs in cancer patients. In this review, we summarize the experimental evidence regarding the effects of green tea-derived polyphenols in conjunction with chemotherapeutic drugs on anti-tumor activity, toxicology, and pharmacokinetics. We believe that the combination of multidrug cancer treatment with green tea catechins may improve treatment efficacy and diminish negative side effects.

## 1. Introduction

**Figure 1:**
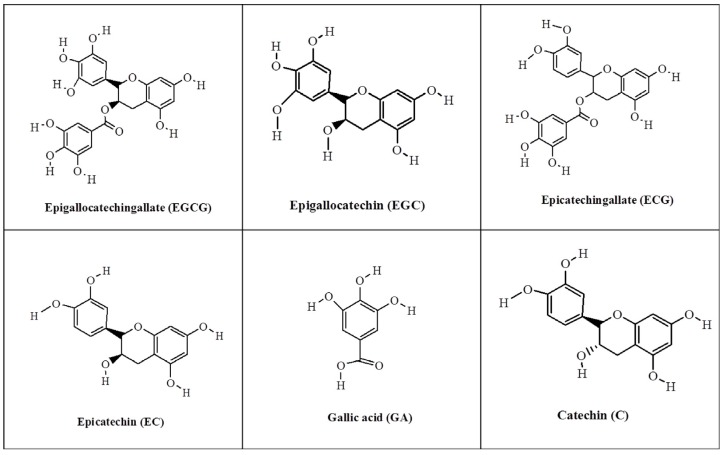
The chemical structures of the main polyphenols in green tea.

Tea made from the plant species *Camellia sinensis* is the most widely consumed beverage other than water. Tea is divided into three subtypes based on fermentation levels: green (unfermented), oolong (partially fermented), and black (highly to fully fermented). Among the various types of tea, green tea is believed to have better antioxidant and health benefits than black and oolong teas [1]. Previous studies reported that green tea could lower the risk of cardiovascular disease, improve brain function, promote fat loss, and combat cancer and type II diabetes, among many other health benefits [2,3,4,5]. Green tea is also associated with many therapeutic effects, including anti-blood coagulation, the reduction of hypertension, oxidative damage repair, HIV treatment, and cancer prevention and treatment [6,7,8,9]. Green tea contains substantial amounts of polyphenols, caffeine, theanine, polysaccharides, and other compounds. Caffeine is a functional alkaloid in tea products. Medicinally it can be used as a cardiac, cerebral, and respiratory stimulant, among other uses. Tea polyphenols are a class of bioactive molecules in green tea, categorized as epistructured catechins or nonepistructured catechins. Epistructured catechins include epicatechin (EC), epicatechin gallate (ECG), epigallocatechin (EGC), and epigallocatechin gallate (EGCG). Nonepistructured catechins include catechin (C), catechin gallate (CG), gallocatechin (GC), and gallocatechin gallate (GCG). EGCG is the most abundant polyphenol in green tea; a typical catechin profile in an extract from green tea leaf is comprised of 10%–15% EGCG, 6%–10% EGC, 2%–3% ECG, and 2% EC. Figure 1 shows the chemical structures of the main polyphenol ingredients in green tea [1,10]. 

Nutritional supplements are commonly integrated into chemotherapeutic strategies for cancer treatment. Combination chemotherapy is an approach to cancer treatment that utilizes multiple medications. This approach can overcome the disadvantages of monotherapy and enhance therapeutic effects in cancer treatment [11]. Due to their various health benefits, green tea polyphenols are increasingly used for cancer prevention or as an adjuvant in chemotherapy. Previous studies have demonstrated that combining chemotherapeutic drugs with green tea could reduce cancer risk, improve survival rates among cancer patients, and decrease chemotherapy-associated side effects [12,13,14,15].

In this paper, we mainly reviewed the experimental data regarding the effects of tea polyphenols in conjunction with chemotherapeutic drugs on anti-tumor activity, toxicology, and pharmacokinetics. We believe that the combination of green tea catechins and anticancer drugs may enhance cancer treatment efficacy and diminish negative side effects. 

## 2. Synergistic Anticancer Activity of Tea Polyphenols and Chemotherapeutic Agents

The combination of green tea catechins and anticancer drugs is a new treatment strategy that has been widely accepted by cancer researchers [11]. Although anticancer drugs and tea polyphenols are very different in terms of structure and function, tea polyphenols can synergistically enhance the effects of anticancer drugs and make them 10–15 times more effective than monotherapy [11]. Some studies have also reported beneficial effects of EGCG or green tea extract with anticancer drugs, such as bleomycin, cisplatin, tamoxifen, and bortezomib [16,17,18,19]. We have also studied the effect of green tea extract on 5-fluorouracil (5-FU) in cancer cells and animals. Our results demonstrated that green tea catechins with anticancer agents are more effective than monotherapy [15]. The effects of tea polyphenols or tea extracts on the therapeutic efficacy of anticancer agents are listed in Table 1.

### 2.1. Combination of Tea Polyphenols and Bleomycin

Bleomycin is frequently used in the treatment of various cancers [10]. However, the monotherapy strategy has often failed to produce therapeutic benefit due to multidrug-resistant cancer. Green tea polyphenols have been used as an adjuvant in bleomycin therapy. Alshatwi et al. [10] reported a synergistic anticancer effect with a combination of tea polyphenols and bleomycin. Various concentrations of tea polyphenols, bleomycin, or tea polyphenols combined with bleomycin were added to cervical cancer cells (SiHa), and then the cell growth, intracellular reactive oxygen species, poly-caspase activity, early apoptosis and expression of caspase-3, caspase-8, caspase-9, Bcl-2, and p53 were observed. This study showed that tea polyphenols combined with bleomycin synergistically inhibited cervical cancer cell viability and proliferation through the induction of apoptosis. Other studies have also suggested that tea polyphenols may increase antitumor activity of bleomycin [18].

### 2.2. Combination of Tea Polyphenols and Cisplatin

Cisplatin is often the first chemotherapeutic agent used to treat many forms of cancer. Unfortunately, cisplatin resistance often develops during the course of treatment. Both preclinical and clinical studies have shown that multiple mechanisms drive tumor resistance to cisplatin. The synergistic effect of cisplatin and tea polyphenols has been studied in vitro and in vivo [20,21,22]. Tea polyphenols combined with cisplatin can decrease proliferation and induce apoptosis in breast cancer cells. Additionally, tea polyphenols plus cisplatin may minimize or slow the development of drug resistance, which may also reduce drug toxicity and improve therapeutic efficacy [18]. The combination of EGCG with cisplatin has increased beneficial effects on cell cycle arrest, modulation of ROS- and apoptosis-related gene expression and potent antioxidant activity when compared with monotherapy. EGCG may also reduce oxidative stress, inhibit proliferation, and sensitize ovarian cancer cells to cisplatin. The combination of tea polyphenols and cisplatin can synergistically inhibit the growth of various cancer cells, such as MCF-7 breast cancer cells and non-small cell lung cancer (NSCLC) A549 cells. Additionally, we found that, compared with cisplatin monotherapy, the combination of cisplatin and EGCG can significantly decrease tumor size in animal models—the data will be reported in the near future.

### 2.3. Combination of Tea Polyphenols and Ibuprofen

Ibuprofen is a non-selective nonsteroidal anti-inflammatory drug (NSAID) [23], which may inhibit the growth of prostate cancer cells in both in vitro and in vivo xenograft models. The synergistic effect of EGCG and ibuprofen (EGCG+ibuprofen) treatment on DU-145 prostate cancer cells has been investigated. This study showed that EGCG + ibuprofen treatment resulted in greater growth inhibition than ibuprofen or EGCG alone. EGCG + ibuprofen treatment acts synergistically to block proliferation and promote apoptosis in DU-145 prostate cancer cells [23].

### 2.4. Combination of Tea Polyphenols and Tamoxifen

Tamoxifen is an anti-estrogenic compound used for the prevention of breast cancer. Green tea is often used as a supplement in breast cancer treatment and prevention [4]. Co-administration of green tea and tamoxifen improves experimental outcomes in breast cancer cell lines and animal models. Green tea increased the inhibitory effect of tamoxifen on the proliferation of estrogen receptor-positive MCF-7, ZR75, and T47D human breast cancer cells in vitro [24,25]. The combination of EGCG (75 and 100 μM) and tamoxifen (5–200 μM) significantly increased apoptosis in PC-9 cells compared to EGCG or tamoxifen alone [26]. When MCF-7 xenograft-bearing mice were treated with both green tea and tamoxifen, their tumor sizes were significantly diminished, and more cancer cell apoptosis occurred in tumor tissue [27,28].

### 2.5. Combination of Tea Polyphenols and Bortezomib

Bortezomib exerts its antitumor effects by reversibly blocking the 26S proteasome [29]. EGCG interferes with bortezomib’s anticancer activity [30]. EGCG’s negative impact on bortezomib efficacy was concentration-dependent in CWR22 xenograft-bearing breast cancer mice. Only very high levels of EGCG antagonized bortezomib’s antitumor activity, while low levels of EGCG had no adverse effects in CWR22 mice [31]. This example demonstrates the negative interaction of EGCG and an anticancer drug.

### 2.6. Combination of Tea Polyphenols and Other Anticancer Drugs

Tea extract or tea polyphenols also synergistically enhance the anticancer activity of other chemotherapy drugs, such as Paclitaxel, sulindac, celecoxib, curcumin, luteolin, docetaxel, retinoids, and so on [32,33,34,35]. Our group has studied the effects of green tea extract and 5-fluorouracil treatment in SW480, BIU-87, and BGC823 human cancer cell lines; a daily dose of green tea (equivalent to <6 cups daily in humans) did not alter the cytotoxicity of 5-FU treatment in these cells [15]. 

### 2.7. Combination of Caffeine and Anticancer Drugs

Caffeine is another ingredient in tea. Caffeine can inhibit the activities of both ATM and ATR—two important protein kinases involved in DNA damage-induced cell cycle arrest and apoptosis. It has been reported that caffeine increased the cisplatin-induced apoptosis in both HTB182 and CRL5985 lung cancer cells by inhibiting ATR and inducing ATM activation [45]. Caffeine could enhance the antitumor effect of cisplatin; when the dosing period of caffeine was increased, the synergistic effect was increased in osteosarcoma-bearing rats [46]. Significant inhibition of tumor growth and prolongation of survival time were also found in sarcoma-bearing mice [47]. Caffeine significantly decreased mutagenicity of the anticancer aromatic drugs daunomycin, doxorubicin, and mitoxantrone. Caffeine decreased the anticancer drug vinblastine-induced chromosomal aberrations and mitotic index in bone marrow cells [48].

## 3. Ameliorating Toxicity Induced by Chemotherapeutic Agents

Two major problems in cancer chemotherapy are adverse side effects and multidrug resistance. Chemotherapy can cause fatigue, nausea, vomiting, and more serious side effects in cancer patients. Previous studies found that anticancer drugs caused serious adverse effects via antioxidant defense abnormalities against reactive oxygen species (ROS) [5,49]. Antioxidants may protect against chemotherapy-induced toxicity. Due to their antioxidant and ROS-scavenging properties, green tea polyphenols could circumvent the adverse effects of ROS and chemotherapy and enhance treatment efficacy (Table 2). Additionally, P-glycoprotein (P-gp) plays an important role in multidrug resistance [50]. EGCG was found to inhibit the transport activity of P-gp and may be an effective P-gp modulator [51]. EGCG also increased chemotherapy drug accumulation in multidrug resistant cells.

Doxorubicin is a potent broad-spectrum chemotherapeutic agent. However, the clinical use of doxorubicin has been seriously limited by its undesirable side effects, especially dose-dependent myocardial injury, which can lead to lethal congestive heart failure [52]. Treatment with green tea ameliorated the cardiotoxicity of doxorubicin. Doxorubicin-induced oxidative stress, heart and liver morphological changes, and metabolic disorders were also mitigated by green tea in male Wistar rats [53]. The mechanism underlying these effects is currently unknown, but it may involve the modulation of enzymes required for lipid synthesis, such as HMG-CoA (3-hydroxy-3-methylglutary-coenzyme A) reductase [54].

## 4. Pharmacokinetic Effect on Chemotherapeutic Agents

Based on food–drug interactions, green tea polyphenols may affect the expression or activities of drug-metabolizing enzymes and drug transporters [55,56]. It is currently unknown whether green tea consumption will alter the pharmacokinetics and bioavailability of a chemotherapeutic agent in cancer patients. Alterations in the pharmacokinetic parameters may also alter the drug’s efficacy or toxicity [57]. Therefore, anticancer drugs should contain warnings on the potential pharmacokinetic interaction of drugs and EGCG. 

In our previous study, we reported that green tea extracts increase the bioavailability of 5-FU in rats [15]. The maximum plasma concentrations (C_max_) and the area under the plasma concentration-time curves (AUC) of 5-FU in rats increased significantly following administration of green tea extract for 14 days. The half-life of 5-FU in plasma was also substantially prolonged [19]. Green tea may decrease the activity of dihydropyrimidine dehydrogenase (DPD)—the initial and rate-limiting enzyme of 5-FU metabolism. Reduced DPD activity may result in decreased 5-FU metabolism, leading to higher plasma concentrations. 

Co-administration of EGCG and irinotecan (CPT-11) altered the pharmacokinetics of CPT-11 and its metabolite, SN-38, in Sprague–Dawley rats [58]. When the animals were pretreated with EGCG, the CPT-11 and SN-38 AUC in plasma were increased by 57.7% and 18.3%, respectively, while the AUC in bile were decreased by 15.8% and 46.8%, respectively. Therefore, the plasma-to-bile distribution ratio (AUC_bile_/AUC_plasma_) was significantly reduced, while the half-lives of CPT-11 and SN-38 in plasma were substantially prolonged. EGCG may inhibit the transport of CPT-11 and SN-38 into the biliary tract by modulating P-gp and reduce hepatobiliary excretion of CPT-11 and SN-38. The increased plasma concentrations of CPT-11 and SN-38 may be associated with enhanced pharmacological effects or toxicity. 

Sunitinib is a novel oral antitumor agent. Plasma concentrations of sunitinib in rats significantly decreased with co-administration of EGCG [59]. The related pharmacokinetic parameters of plasma sunitinib (such as AUC_0–∞_ and C_max_) were markedly reduced by the co-administration of EGCG. In the sunitinib with EGCG group, the mean C_max_ decreased by 47.7% compared with the sunitinib with water group, while AUC_0–∞_ significantly decreased by 51.5%. These results indicate that EGCG markedly reduced the bioavailability of sunitinib. Therefore, it is necessary for patients receiving sunitinib therapy to avoid consuming green tea or EGCG dietary supplements.

## 5. Human Trials

The aims of the clinical trials are to study the effectiveness of green tea extract in treating cancer patients. A total of 100 clinical trials involving both green tea and cancer are listed in clinicalTrials.gov [60]. Some results proved that green tea contains ingredients that may prevent or slow the growth of certain cancers. For example, in the clinicalTrial NCT00685516 (a multicenter, randomized, phase II trial), 113 men diagnosed with prostate cancer were randomized to consume six cups daily of brewed green tea, black tea, or water (control) prior to radical prostatectomy. The prostate tumor markers of cancer development and progression were determined by tissue immunostaining of proliferation (Ki67), apoptosis (Bcl-2, Bax, Tunel), inflammation (nuclear and cytoplasmic nuclear factor kappa B (NFκB)) and oxidation (8-hydroxydeoxy-guanosine (8OHdG)). Blood and urine samples, as well as tissue from diagnostic biopsy and radical prostatectomy specimens were evaluated by high performance liquid chromatography and ELISA analysis; the concentrations of total and free tea polyphenols (i.e., EGCG, EC, EGC, and ECG), theaflavins, and conjugated/colonic tea metabolites were also detected [61]. The estimated study completion date is August 2017; some primary data have been published [62]. The results showed that green tea can change NFκB and systemic oxidation, and future longer-term studies are warranted to further examine the role of green tea for prostate cancer prevention and treatment.

## 6. Conclusions

The benefits of combining tea polyphenols with anticancer compounds are now widely accepted by cancer researchers. Previous studies have demonstrated that a combination of chemotherapeutic drugs and green tea extract could enhance therapeutic effects and reduce the adverse side effects of anticancer drugs most of the time. Several papers have also reported the potential for negative interactions between tea polyphenols and anticancer drugs. In this article, we provided a brief overview of the pharmacodynamics, toxicology, and pharmacokinetic interactions between green tea and anticancer drugs. We believe that the combination of green tea and anticancer drugs may be important in enhancing therapeutic efficacy while diminishing negative side effects.

## Figures and Tables

**Table 1:** The effects of green tea catechins on anticancer compounds in anti-tumor activity.

Anticancer Drugs	Experiment	Effects	Reference
Bleomycin	SiHa cervical cancer cells or uterine cervical cancer cells were treated with tea polyphenol and bleomycin; poly-caspase activity, early apoptosis, and the expression of caspase-3, caspase-8, caspase-9, Bcl-2, and p53 were assessed.	Synergistic increase in antitumor effects.	[10]
5-Fluorouracil (5-FU)	Some cancer cells—such as human SW480, BIU-87, BGC823, and Hep3B—were treated with green tea and 5-FU; the cytotoxicity, cell apoptosis, and proliferation were studied.	Increase in cell apoptosis; synergistic inhibition of cell proliferation; no reduction in antitumor activity.	[15,35]
Cisplatin	Cancer cells YCU-N861, YCU-H891, Hep3B, SW480, BIU-87, BGC823, et al. were coadministered cisplatin with tea polypnenols; the cell apoptosis and proliferation were studied.	Synergistic inhibition of cell proliferation; induction of apoptosis.	[20,21,22]
Ibuprofen	DU-145 cells were treated with EGCG and ibuprofen; cell death analysis, immunoblotting, RT-PCR analysis, and caspase activity assay were used.	Synergistic effect on the anti-proliferative and pro-apoptotic action.	[23]
Tamoxifen	Cancer cells PC-9, MCF-7, and MDA-MB-231were treated with tea polyphenols and tamoxifen; some factors such as EGFR, MMP-2, MMP-9, and EMMPRIN were assessed.	Induction of apoptosis; enhanced expression of apoptotic genes; synergistic increase in antitumor effects.	[24,25,26]
Sulindac	PC-9 cancer cells were treated with sulindac and tea polyphenols; gene expression was assessed.	Induction of apoptosis; enhanced expression of apoptotic genes.	[27,28]
Bortezomib	Cancer cells 26S and CWR22 were treated with bortezomib and tea polyphenols; cell apoptosis and proliferation were assessed.	Antagonized antitumor activity.	[29,30,31]
Celecoxib	A549 and MCF-7 cancer cells were treated with celecoxib and tea polyphenols; the cell activity and gene expression were assessed.	Increased cell apoptosis; enhanced expression of GADD153 gene	[32]
Luteolin	Cancer cells H292, A549, H460, and Tu212 were treated with luteolin and EGCG; phosphorylation of p53 was studied.	Induction of caspase-8 and caspase-3 cleavage; increase in cell apoptosis.	[33]
Docetaxel	PC-3ML cancer cells were treated with docetaxel and tea polyphenols; hTERT and Bcl-2 were studied.	Increase in the expression of apoptotic genes; reduction in growth rate of cancer cells.	[34]
Curcumin	Cancer cells PC-9, A549, NCI-H460, and ER alpha-breast cancer cells were treated with curcumin and tea polyphenols; the cell activity and cell cycle were assessed.	Induction of apoptosis; enhancement of cell cycle arrest at G1 and S/G2 phases.	[36,37,38]
Quercetin	Cancer cells PC-3, LNCaP, and CWR22Rv1 were treated with quercetin and tea polyphenols; the cell growth and gene expression were assessed.	Synergistic expression of androgen receptor; inhibition of cancer cell growth.	[39,40]
Paclitaxel	PC-3ML cancer cells were treated with paclitaxel and tea polyphenols; the cell growth and apoptotic gene expression were assessed.	Increase in the expression of apoptotic genes; reduction in growth rate of cancer cells.	[34,41]
Doxorubicin	Cancer cells BEL-7404/DOX, PC-3ML, IBC-10a, and PCa-20a were treated with doxorubicin and tea; the cell proliferation and apoptosis were assessed.	Enhanced sensitivity to doxorubicin; synergistic increase in antitumor effects.	[42]
Resveratrol	Cancer cells ALVA-41, PC-3, and MCF-7 were treated with resveratrol and green tea; the cell growth and apoptosis were assessed.	Inhibition of cell growth; induction of apoptosis	[19,43]
Sulforaphane	Cancer cells PC-3 AP-1, HT-29, SKOV-ip1, SKOVTR-ip2 were treated with sulforaphane and EGCG; the cell activity and gene expression were assessed.	Diminished induction of cancer cell activity; inhibition of cell viability; increase in apoptosis.	[34,44]

EGFR (epidermal growth factor receptor); MMP-2, MMP-9 (a family of matrix metalloproteinases); EMMPRIN (extracellular matrix metalloproteinase inducer); hTERT (human telomerase reverse transcriptase); ER (estrogen receptor).

**Table 2:** A combination of green tea catechins and anticancer compounds ameliorating the toxicity induced by chemotherapeutic agents.

Anticancer Drugs	Experiment	Effects	Reference
Doxorubicin	Wistar albino rats with cardiotoxicity induced by doxorubicin were treated with green tea. AST, CK, LDH, LPO, cytochrome P450, blood glutathione, tissue glutathione, and enzymatic and non-enzymatic antioxidants were evaluated along with histopathological studies.	Oral administration of green tea prevented doxorubicin-induced cardiotoxicity by accelerating heart antioxidant defense mechanisms and downregulating the LPO levels to the normal levels.	[52]
Doxorubicin (DOX)	Neonatal Rats with cardiotoxicity induced by doxorubicin were treated with EGCG; LDH, MnSOD, catalase, and glutathione peroxidase were detected.	EGCG could protect cardiomyocytes from DOX-induced oxidative stress by attenuating ROS production and apoptosis, and increasing activities and protein expression of endogenous antioxidant enzymes.	[53]
Doxorubicin	Rats were treated with doxorubicin and different doses of EGCG. Cardiac enzymes (creatine kinase isoenzyme-MB and lactate dehydrogenase) and histopathological changes were studied.	EGCG possesses cardioprotective action against doxorubicin-induced cardiotoxicity by suppressing oxidative stress, inflammation, and apoptotic signals, as well as the activation of pro-survival pathways.	[54]

AST (aspartate transaminase); CK (creatine kinase); LDH (lactate dehydrogenase); LPO (lipid peroxidation); MnSOD (superoxide dismutase).
